# Unusual signals in a halo catheter: what is the mechanism?

**DOI:** 10.1016/s0972-6292(16)30459-4

**Published:** 2012-01-31

**Authors:** Jane Caldwell, Rodrigo Miranda, Damian Redfearn, Adrian Baranchuk

**Affiliations:** Division of Cardiology (Arrhythmia Service), Kingston General Hospital, Queen's University, Kingston, Ontario, Canada

**Keywords:** atrial flutter, functional and anatomical conduction block

## Abstract

In this "featured arrhythmia" article we present a set of unusual intracardiac electrode tracings that were recorded in a patient with typical clockwise flutter but a very dilated right atrium. The potential mechanism underlying this phenomenon is discussed with reference to the current literature.

## Case Presentation

**Figure 1:**
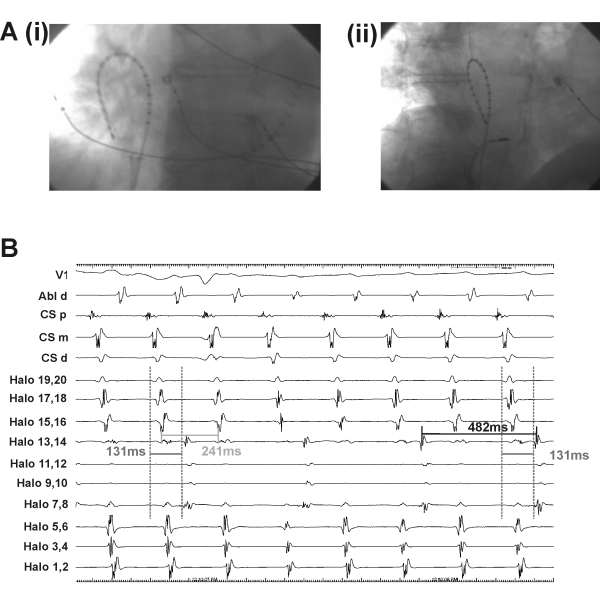
Panel A: Fluroscopy of catheter placement in (i) LAO projection and (ii) RAO projection. Panel B: Intracardiac electograms from the RA during typical counter-clockwise atrial flutter with a cycle length of 241 ms (light grey callipers). The activation fails to propagate into the myocardium underlying dipole of Halo 7-8 to 13-14 on every flutter cycle. Activation in this area has cycle length 482 ms (black callipers). When propagation occurs, there is a stable delay (dark grey dotted line callipers, 131ms) (CS = coronary sinus, Abl ablation catheter, d - distal, p - proximal, m - middle, H20 most proximal halo pole to H1 the most distal).

**Figure 2:**
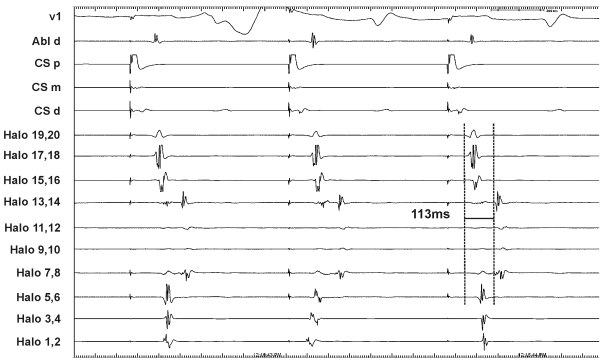
Intracardiac electograms from the RA during pacing from coronary sinus electrode dipoles 9-10. Delayed activation of Halo dipoles 7-8, 13-14 remains but with 1:1 propagation of activation (CS = coronary sinus, Abl ablation catheter, d - distal, p - proximal, m - middle, H20 most proximal halo pole to H1 the most distal).

An 81 year old gentleman with known hypertension, COPD, obstructive sleep apnea requiring nocturnal CPAP and persistent atrial flutter was admitted electively for catheter ablation. Echocardiography prior to the procedure revealed a moderately dilated right atrium (volume index 40ml/m^2^) and mild left ventricular hypertrophy with preserved systolic function. The 12-lead electrocardiogram was consistent with typical isthmus-dependent counter-clockwise (CCW) flutter. Under fluoroscopic guidance in the LAO projection, a steerable decapole catheter was placed in the coronary sinus, an adjustable duodecapole catheter placed around the tricuspid valve annulus and an ablation catheter placed on the cavotricuspid isthmus at the 6 o'clock position (Figure 1Ai). All catheters were introduced via the right femoral vein. Initial recordings from the halo catheter during atrial flutter showed an area of myocardium with: (a) delayed conduction compared to the adjacent dipoles and (b) apparent 2:1 conduction block (Figure 1B). Fluoroscopy in the RAO projection highlighted the relatively posterior placement of the halo catheter compared to the coronary sinus. On attempts at entrainment, the atrial flutter cardioverted to sinus rhythm after a brief episode of atrial fibrillation. In sinus rhythm, and during slow rate pacing from the coronary sinus, the conduction delay remained (Figure 2). What is the mechanism of underlying these recordings?

## Discussion

There are three potential explanations for the intracardiac electogram pattern observed in Figure 1B; (i) dual tachycardia, (ii) artifact or (iii) functional conduction block. Dual tachycardia within the same chamber is unusual but has been documented in the left ventricle[1]. Glover et al demonstrated how the faster tachycardia could transiently entrain the slower tachycardia but for most of the time there was no inter-relationship between the 2 tachycardias. In contrast, in the case presented here, the time delay between the first activation in the septal halo poles (Halo19-20) and the first activation in the "delayed" area (Halo 7-8) was constant at ~131ms and the cycle length in the "delayed" area (482ms) was double the flutter cycle length (241ms) (Figure 1B). This regularity makes dual tachycardia unlikely. Whilst the relatively posterior placement of the halo catheter in an enlarged RA (Figure 1Aii) could result in a less stable position, it is unlikely that intermittent contact with the myocardium is the mechanism involved here. Whilst intermittent contact could give a false impression of conduction block it is unlikely that it would give such a tightly repetitive pattern (Figure 1B).

Functional conduction block within the crista terminalis (CT) during typical atrial flutter has been well documented [2]. In these studies the double potentials visualised in the catheters overlying the CT were felt to represent collision between delayed and non-delayed activation fronts [2]. This functional block within the CT and Eustachian ridge acts as the posterior barrier for the re-entrant flutter circuit. However, it is unlikely "conventional" CT functional block alone could account for activation patterns observed in the case reported here, as (a) functional CT block is associated with 1:1 conduction in flutter [2] and (b) the double potentials are rarely persistent in sinus rhythm or slow atrial pacing [2]. In contrast, in the case reported here, 2:1 block was observed during flutter and was associated with conduction delay not only during flutter but also in sinus rhythm and slow rate pacing (Figure 2). One possible explanation for this could be a complex "figure of 8" re-entry mechanism involving conduction on both sides of the crista terminalis (i.e. posteriorly as well as anteriorly). As identical delay in conduction over the crista was seen after successful CTI ablation this makes this mechanism unlikely.

Persistent conduction delay has been demonstrated in fixed anatomical conduction block, such as that observed in the canine right atrial crush model of atrial flutter [3] or in inter-atrial block [4]. In this report, Irie et al demonstrated a case of isthmus-dependant CCW atrial flutter associated with an atypical electrogram morphology due to the presence of persistent conduction block in the proximal coronary sinus (CS) [4]. This conduction block forced left atrial activation to occur through alternative routes such as Bachman's bundle, the interatrial septum or both, and hence the atypical electrogram morphology. In the case presented here the electrogram was typical of CCW flutter, but the persistent conduction delay observed in sinus rhythm and slow CS pacing imply a degree of fixed anatomical conduction block. Functional block would be expected to improve at longer cycle lengths when the source to sink mismatch is not so large. Thus, this observation must be associated with a structural barrier such as the scar tissue associated with the grossly dilated right atrium. Catheter ablation at the CTI is appropriate and was performed.
